# CHiCP: a web-based tool for the integrative and interactive visualization of promoter capture Hi-C datasets

**DOI:** 10.1093/bioinformatics/btw173

**Published:** 2016-04-08

**Authors:** E. C. Schofield, T. Carver, P. Achuthan, P. Freire-Pritchett, M. Spivakov, J. A. Todd, O. S. Burren

**Affiliations:** ^1^JDRF/Wellcome Trust Diabetes and Inflammation Laboratory, NIHR Cambridge Biomedical Research Centre, Department of Medical Genetics, Cambridge Institute for Medical Research, University of Cambridge, Cambridge CB2 0XY, UK; ^2^Nuclear Dynamics Programme, The Babraham Institute, Cambridge CB22 3AT, UK

## Abstract

**Summary:** Promoter capture Hi-C (PCHi-C) allows the genome-wide interrogation of physical interactions between distal DNA regulatory elements and gene promoters in multiple tissue contexts. Visual integration of the resultant chromosome interaction maps with other sources of genomic annotations can provide insight into underlying regulatory mechanisms. We have developed *C*apture *HiC P*lotter (CHiCP), a web-based tool that allows interactive exploration of PCHi-C interaction maps and integration with both public and user-defined genomic datasets.

**Availability and Implementation:** CHiCP is freely accessible from www.chicp.org and supports most major HTML5 compliant web browsers. Full source code and installation instructions are available from http://github.com/D-I-L/django-chicp.

**Contact:** ob219@cam.ac.uk

## 1 Introduction

A large majority of single nucleotide variations (SNVs) associated with human disease lie outside of known coding regions and are enriched in DNase I hypersensitive regions (Maurano *et al.*, 2012). Promoter capture Hi-C enables the detection of physical chromatin interactions between gene promoter regions and distal DNA gene expression regulatory elements, globally and at high resolution (Belton *et al.*, 2012; Sahlen *et al.*, 2015; Schoenfelder *et al.*, 2015). By integrating with genome-wide association data, it may be possible to gain further insight into the genetic mechanisms underlying a particular trait or disease (Mifsud *et al.*, 2015). However, owing to a dependence on restriction enzymes in the underlying method, defined interactions are based on specific restriction fragment intervals. The resultant genomic interaction maps are therefore complex, as a single promoter fragment can interact with multiple distal restriction fragments of heterogeneous size, and interactions may vary across different tissue contexts. Current genome browsers with capabilities for the visualization of these interactions, such as WashU Epigenome Browser (Zhou *et al.*, 2013) and Ensembl (Cunningham *et al.*, 2015), provide a linear view which can limit a user’s ability to resolve these complex interaction patterns and how they relate to other genomic features of interest. A circular representation can be helpful in this context, but existing circular viewers, such as J-Circos (An *et al.*, 2015), CircOS (Krzywinski *et al.*, 2009), GView (Petkau *et al.*, 2010) and GenomeD3Plot (Laird *et al.*, 2015), are not specifically designed and optimized to allow interactive and integrative visualization of human PCHi-C datasets.

CHiCP employs a hybrid approach: users see a circular overview for all interactions for a given SNV, gene or region, with the option to highlight a particular interaction of interest that results in a standard linear view. We demonstrate its functionality by integrating a recent promoter capture Hi-C dataset (Mifsud *et al.*, 2015) with publically available GWAS summary statistics (http://www.immunobase.org), Ensembl gene annotations (Cunningham *et al.*, 2015) and Roadmap epigenomics chromatin segmentation states (Roadmap Epigenomics *et al.*, 2015).

## 2 Methods

We obtained PCHi-C data for GM12878 and CD34^+ ^cells (Mifsud *et al.*, 2015), detected interactions in these data using the CHiCAGO pipeline (Cairns *et al.*, 2015) and converted the WashU Browser formatted output files into tab-delimited format using a custom Perl script. We downloaded chromatin segmentation states from the Epigenomic Roadmap web portal (http://www.ncbi.nlm.nih.gov/geo/roadmap/epigenomics/), Gene annotations from Ensembl v75 (ftp://ftp.ensembl.org/pub/release-75/gtf/homo_sapiens/Homo_sapiens.GRCh37.75.gtf.gz) and autoimmune disease summary association statistics from ImmunoBase v1.11 (http://www.immunobase.org). We processed all datasets using Elasticsearch (http://www.elastic.co) to obtain indexed object stores and developed a RESTful web application using the Django (v1.8) framework (https://djangoproject.com) to facilitate querying and data integration. We developed a browser-based interactive visualization using D3.js (http://d3js.org/) and jQuery JavaScript (http://jquery.com/) libraries to provide an asynchronous visual interface to the underlying Django web application.

## 3 Results

**Fig. 1.**
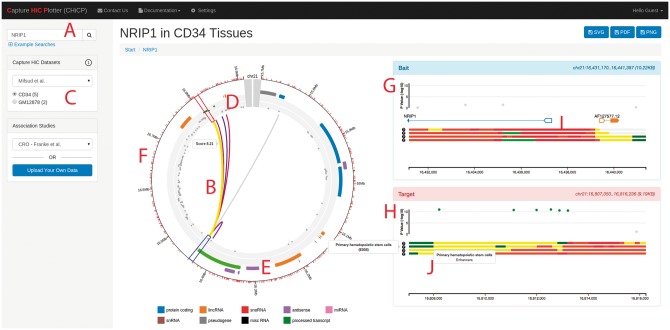
Typical display from CHiCP showing *NRIP1* in CD34+ cells integrated with Franke *et al.* (2010). Crohn’s disease GWAS summary statistics. (**A**) Search box for gene, reference SNP (rs) ID or genomic location; (**B**) interaction between *Hind*III fragments; (**C**) PCHiC datasets and tissues for selection; (**D**) association summary statistics; (**E**) gene type with colour depicting biotype; (**F**) genomic scale and *Hind*III cleavage sites; (**G**) linear representation of Bait fragment; (**H**) linear representation of the target fragment; (**I**) canonical genes with transcription start sites shown as outlined exons; (**J**) epigenetic cell states for selected tissue (*yellow* – enhancers; *dark green* – weak transcription; *light green* – transcription at gene 5′ and 3′; *red* – flanking active TSS)

The user interface and visualization of CHiCP consists of a search toolbox on the left-hand side of the browser window (Fig. 1A) that allows searching of the configured PCHi-C and association statistics datasets by gene name, Ensembl gene id, Reference SNP (rs) ID or a genomic region (e.g. chr21:16165420-16959712). Tissue context can be altered by using radio buttons labeled with available tissue types followed, in brackets, by the number of significant interactions for that type. Finally, the ‘Association Study’ drop-down menu allows users to integrate either public or private genetic association summary statistics. The data visualization is split into three panels, a central navigation panel showing a circularized overview of interactions and annotations for current search term and two right-hand ‘detail’ panels. From the centre of the circular plot, matching interactions from the selected tissue context are shown as coloured arcs connecting restriction fragments (Fig. 1B). Moving outwards, association summary statistics are shown in a circularized Manhattan plot, by default genome-wide significant associations (*P* < 5 × 10^−8^) are shown in green (Fig. 1D). Genes are coloured according to biotype (Fig. 1E). Finally, a genomic scale is displayed with red tick marks detailing the positions of restriction enzyme fragments (Fig. 1F). Clicking on an interaction opens a linear representation of the bait and target regions in separate panels on the right-hand side (Fig. 1G, H), with genes shown in canonical format, and outlined exons flagging transcription start sites (Fig. 1I). Where data are available, extra tracks showing epigenetic states of the cells are shown (Fig. 1J). Buttons in the top bar allow snapshotting of the current view for the purposes of sharing and figure generation.

We used CHiCP to not only validate the findings of Misfud *et al.* that *NRIP1* is a likely causal gene in ulcerative colitis (UC), but additionally, that UC genome-wide significant variants from Franke *et al.* (2010) overlap CD34^+ ^specific enhancer regions (Fig. 1).

## 4 Discussion

We present CHiCP as an innovative and powerful web-based tool for the interactive and integrative visualization of human PCHi-C data with other genomic and genetic datasets. We provide a demonstration of its utility by focusing on the integration of a set of autoimmune disease genetic association summary statistics with a published PCHi-C dataset of relevant cell types. As more PCHi-C datasets across multiple tissue types enter the public domain, visualization tools such as ours will have increasing utility in making these results accessible in the context of other relevant genomic annotations. It might also provide a platform for creating a browsable PCHi-C compendium that has the possibility to facilitate the understanding of the role of regulatory variation in human disease in specific tissue contexts. Indeed, future work will examine the feasibility of adding functionality for users to add their own public and private interaction datasets as well as further modifications to allow the general visualization of other sources of high resolution Hi-C data (e.g. enhancer capture). Future user interface improvements might allow for a more exploratory approach at the genome-wide scale. The software is freely available, is implemented using standard open source web components, and thus can be modified to support diverse use cases and datasets both publicly and privately.
